# Personality variables in risk perception, learning and risky choice after safety training: Data of two empirical intervention studies contrasting immersive VR and PowerPoint

**DOI:** 10.1016/j.dib.2018.08.153

**Published:** 2018-08-30

**Authors:** Johannes Leder, Tina Horlitz, Patrick Puschmann, Volker Wittstock, Astrid Schütz

**Affiliations:** a Department of Psychology, University of Bamberg, 96047 Bamberg, Germany; b Institute for Machine Tools and Production Processes, Technische Universität Chemnitz, Reichenhainer Str. 70, 09126 Chemnitz, Germany

## Abstract

The data provided contains personality variables as well as risk perception, choice data and learning outcomes after safety training which differed between subject regarding whether it was carried out in immersive virtual reality (VR) or as a PowerPoint. The data presented here is reported in the study of Leder et al. (in press) [3] where the impact of presentation medium on safety training effectiveness was investigated.

The data is hosted on the open science project: https://osf.io/rxq5w/?view_only=cd42aa4b97514f16af8884697450d2a6.

**Specifications Table**

**Table:** 

Subject area	*Psychology, Ergonomics*
More specific subject area	*Effectiveness of safety training influenced by presentation medium, personality, risk perception*
Type of data	*Table (csv file)*
How data was acquired	*Experimental study and questionnaire*
Data format	*Raw and aggregate*
Experimental factors	*Two groups between subjects design: one group received safety training via immersive virtual reality the other group via PowerPoint.*
Experimental features	*Intervention study with Pre-Post Test Design.*
	*Assessment of long-term effects in study 2.*
Data source location	*Chemnitz, Germany*
Data accessibility	*Data is available at*: https://osf.io/rxq5w/?view_only=cd42aa4b97514f16af8884697450d2a6

**Value of the data**

• Personality and risk perception of a hazardous machine can be investigated.
• Personality and its effect on risky choices can be investigated.
• Personality and the effect of safety training can be assessed.
• Data can be compared to existing studies testing the effectiveness of virtual reality instructions in safety training.

## 1 Data

The data shared consist of self-reports and decisions. The variables reflect risk perception, learning, risky choice, sense of presence and personality variables (control, conscientiousness, domain-specific risk attitudes). To ensure perfect anonymity of the participants, participant׳s age and education were removed from the publicly shared data. The participants are high school students and their mean age was 18 years.

Data from two independent experiments is provided findings are discussed in Leder et al. (in press). The csv files contain raw data and aggregated data for each experiment. The r-script contains all pre-analysis operations, which were used to derive at the aggregated data.

The *R-file* “Personality variables in risk perception, learning and risky choice after safety training.R” contains operations we carried out prior to analysis. Furthermore, the file contains information about scale construction and references to the scales used. We also provide the labels of all variables in the data sets in the repository.

The *data files* are in a wide format, which means that points of measurement, if there were multiple, are identified by variable name[.]Time (e.g., severity measure at t2=severity.2).

The *variable labels* are provided in a separate file for each study in the repository.

## 2 Experimental design, materials and methods

Both studies were designed as intervention studies, and risk perceptions were assessed before and after the safety training, the sense of presence was assessed during the safety training. Recall of safety knowledge and decision making were measured after the safety training. Study 1 and Study 2 differed in the personality variables that were assessed. Personality variables in Study 1 were locus of control, conscientiousness, and domain-specific risk perception. Personality variables in Study 2 were domain-specific risk attitudes. Furthermore, in Study 2, we tested if differences in risk perception and learning were persistent over time of the training by employing a follow-up assessment after 6 months.

Data were processed in the following way:

– Scales can be reconstructed based on the manuals of the FKK [4], locus of control, NEO-FFI [2] for conscientiousness and DOSPERT [1] for domain specific risk attitudes. A file necessary to aggregate the scales from items is provided together with the references
– We did not use any filtering and did not exclude any participant

Study 1:

Participants (*N* = 53) were randomly assigned either to a safety training carried out based on immersive virtual reality or by power point.

Personality scales measuring locus of control [4], conscientiousness [2] and risk perception [1] were administered. Severity and probability judgments were measured before the safety training and after safety training. Sense of presence was measured during the safety training. After safety training severity and probability judgments were measured, learning outcomes were assessed with a recall test related to the information in the safety instruction. In addition participants were to point out hazards and safety procedure while facing a hazardous machine. Finally, participants were to decide which amount of money they would request in order to work with an unsafe rather than with a safe machine.

Study 2:

Participants (*N* = 70) were randomly assigned either to a safety training carried out based on immersive virtual reality or power point.

Personality scales measuring domain specific risk attitudes [1] were administered. Severity and probability judgments were measured before the safety training, after safety training and six months after safety training. Sense of presence was measured during the safety training. After the safety training learning outcomes were assessed with a recall test. In addition participants were to list hazards and safety procedures when faced with a hazardous machine. Finally, participants were to decide which amount of money they would request in order to work with an unsafe rather than with a safe machine.

The learning outcomes and identified hazards were assessed again six months after the safety training with an online test.
